# Time-dependent plasma metabolic shifts and mechanistic insights into systemic consequences of cerebral ischemia in middle-aged patients

**DOI:** 10.1007/s11306-026-02439-z

**Published:** 2026-04-28

**Authors:** Eva Baranovicova, Jana Dluha, Egon Kurca, Vladimir Nosal, Lucia Babalova, Stefan Sivak, Erika Halasova, Simona Holubcikova, Peter Racay

**Affiliations:** 1 Biomedical Center BioMed, Jessenius Faculty of Medicine in Martin, Comenius University in Bratislava, Mala Hora 4, Martin, 036 01 Slovakia; 2 Clinic of Neurology, Jessenius Faculty of Medicine in Martin, Comenius University in Bratislava, Kollarova 2, Martin, 036 59 Slovakia; 3 Department of Medical Biochemistry, Jessenius Faculty of Medicine in Martin, Comenius University in Bratislava, Mala Hora 4, Martin, 036 01 Slovakia

**Keywords:** Cerebral ischemia, Blood metabolomics, Energy metabolism

## Abstract

**Objectives:**

To investigate how acute ischemic stroke induces systemic metabolic reorganization and to characterize the temporal dynamics of these alterations in relation to stroke severity.

**Methods:**

Blood plasma samples were collected from patients (*n* = 42) with acute ischemic stroke, including individuals undergoing mechanical thrombectomy, at two time points: within 24 h and five days after stroke onset. Analysed were circulating metabolites linked to energy metabolism and the most abundant amino acids.

**Results:**

Stroke triggered rapid and systemic metabolic changes, including altered circulating glucose and lactate levels indicative of shifts in substrate utilization and inter-tissue energy redistribution. Ketone body concentrations were elevated, providing alternative fuels for metabolically stressed tissues. The magnitude and timing of systemic changes, including dynamic modulation of amino acids, were proportional to ischemic severity, demonstrating a graded physiological response.

**Conclusion:**

This study provides a characterization of the dynamic metabolic response to acute ischemic stroke, revealing extensive alterations in energy metabolism, amino acid pathways, and systemic metabolic regulation. Certain exhibited rapid changes that nearly normalized by Day 5, whereas other metabolites, including specific amino acids, showed delayed responses, highlighting the temporal heterogeneity of post-stroke metabolic response. The interconnection of metabolic pathways underscores that post-stroke alterations extend beyond isolated processes, forming a systemic network of biochemical interactions that may affect recovery trajectories and peripheral organ function. These findings provide insight into inter organ-level coordination after stroke and highlight the potential of plasma metabolomic profiling for monitoring systemic responses and recovery following cerebral ischemic events.

## Introduction

Ischemic stroke, which accounts for approximately 85% of all stroke cases, results from the sudden occlusion of cerebral arteries, leading to restricted blood flow, oxygen and glucose deprivation, followed by neuronal injury (Bevers & Kimberly, 2017). The metabolic response to ischemic stroke is rapid and complex, reflecting both the extent of neuronal injury and the body’s attempt to restore homeostasis. The vast majority of studies focus on describing metabolic changes in the early period after the onset of symptoms as reviewed in the following works (Baranovicova et al., 2023; Chumachenko et al., 2022; Shin et al., 2020; Zhang et al., 2022), while monitoring the time course of changes after ischemic cerebral stroke is preferred using animal models of cerebral ischemia. Data from human stroke studies, as well as experimental animal models of cerebral ischemia, suggest that early normalization of energy metabolism after ischemia is a key determinant of subsequent cerebral damage and clinical outcome (Baranovicova et al., 2023; Baranovicova et al., 2020).

Stroke predominantly affects older age groups, yet in very elderly patients, baseline metabolism is already subtly altered due to age-related changes in glucose regulation, mitochondrial function, and oxidative stress (Palmer & Jensen, 2022; Khalaf et al., 2025), which may influence the magnitude and pattern of the post-stroke metabolic response. The purpose of this study was to profile the systemic metabolic response to acute ischemic stroke in patients within an age range of 49–65 years at two time points, with an emphasis on key metabolic pathways, including glycolysis, the tricarboxylic acid (TCA) cycle, ketone body metabolism, and amino acid turnover. Patients who underwent mechanical thrombectomy (MT) and presented with significantly higher NIHSS scores were also analyzed as a separate group. We determined and critically assessed the diagnostic potential of metabolite patterns using receiver operating characteristic (ROC) curve analysis. In summary, this work aimed to elucidate time-dependent systemic metabolic adaptations after ischemic stroke and their potential relevance for pathophysiological understanding and clinical stratification.

## Materials and methods

### Study population

The patients were selected from all collected samples according to the following diagnosis codes: I630 (*n* = 2), I632 (*n* = 3), I633 (*n* = 20), I634 (*n* = 15), I635 (*n* = 2). Blood samples were taken on Day 1 of admission to the Department of Neurology or ICU/CCU, within 24 h of symptom onset, with data labelled Stroke1. The second blood sample for metabolomic analysis was collected on Day 4 (*n* = 4) and Day 5 (*n* = 38), the data were combined into one group and labelled Stroke5. A total of 42 patients with acute stroke were included in the final analysis (19 male / 23 female). The median age was 57 years, with an interquartile range (IQR) of 11.5 years.

Based on available medical records, the patients had the following comorbidities: compensated type 2 diabetes mellitus (DM II) (*n* = 8), previous ischemic stroke (*n* = 8), history of oncologic disease (more than 10 years ago, *n* = 2), ischemic heart disease (*n* = 15), obesity (*n* = 7) and hypertension (*n* = 36). Written informed consent was obtained from each eligible subject who agreed to participate in the study. The biochemical and hematological characteristics of the patients are summarised in Tables 1 and 2. The absence of data on Day 5 is primarily attributable to differences in the timing of sample collection between clinical biochemistry and metabolomic analyses.

Regarding the patients’ medical conditions, no intravenous glucose infusions were administered in the pre-hospital setting. During hospitalization, glucose levels remained consistently above 5 mmol/L across all measured time points (including intermediate points between Day 1 and Day 5), making intravenous glucose administration due to hypoglycemia unlikely; however, this could not be confirmed with certainty due to incomplete treatment records. Patients were not receiving insulin prior to hospital admission. Insulin therapy may have been initiated during hospitalization in cases of documented hyperglycemia (glucose level > 10 mmol/L), observed in a subset of patients (*n* = 8); however, its administration could not be verified due to limited treatment data.

Exclusion criteria included age over 65 years (due to lack of appropriate matched controls and to reduce the impact of age-related metabolic changes, such as altered mitochondrial function, chronic inflammation, comorbidities, enhanced catabolism and others), neurological disorders, active malignancy, advanced chronic liver disease, renal failure (serum Cre > 150 µmol/L), DM I, uncompensated DM II, hepatitis, pancreatitis, acute intoxication. Only patients whose samples could be collected from the hospital and rapidly centrifuged to obtain plasma within one hour (so that the remaining viable blood cells in the tube would not consume glucose, produce lactate, or induce other metabolic changes) were included.

Systemic intravenous thrombolysis was applied in 5 patients. From the total cohort, 12 patients with a moderate to severe NIHSS score (mean 13.5) were identified and analyzed as a distinct subgroup. These patients underwent mechanical thrombectomy (MT), and their outcomes were evaluated both separately and in combination with the overall study population.

**Table 1:** Basal biochemical parameters of Stroke1 patients

	Reference values	*N* total / missing	Median	IQR (Q3 - Q1)
glucose [mmol/L]	4,1–5,9	32/10	7.2*	3.05
creatinine [µmol/L]	59–104	35/7	74	32
total bilirubin [µmol/L]	5,0–21,0	33/9	12.5	8.8
bilirubin konjug [µmol/L]	0,1–3,4	32/10	2.15	1.8
AST [µkat/L]	< 0,85	32/10	0.39	0.13
ALT [µkat/L]	< 0,85	32/10	0.32	0.16

*Parameter that exceeds the reference range

**Table 2:** Blood parameters of the patients included in the study; Stroke1 on Day 1 and Stroke5 on Day 5

		Stroke1			Stroke5		
	Reference values	*N* total / missing	Median	IQR (Q3 - Q1)	*N* total / missing	Median	IQR (Q3 - Q1)
CRP [mg/L]	< 5,0	28/14	4.6	13	NA *		
WBC [10^9^/L]	4,00–10,00	42/0	8.4	3.2	35/7	7.2	2.6
RBC [10^12^/L]	3,50 − 5,50	42/0	4.42	0.5	35/7	4.17	0.74
HGB [g/L]	110–160	42/0	137	24	35/7	123	21
HCT [%]	37–52	42/0	41	7	35/7	38	5
MCV [fL]	80,0-100,0	42/0	91.7	7.7	35/7	91.5	10.2
MCH [pg]	27,0–34,0	42/0	30.2	3.4	35/7	30.3	3.9
MCHC [g/L]	320–360	42/0	331	8	35/7	332	12
RDW [%]	11–16%	42/0	14.2	2.2	35/7	14.5	2
PLT [10^9^/L]	100–300	42/0	229	89	35/7	218	97
MPV [fL]	6,5–12,0	41/1	8.7	1.7	35/7	9.35	1.05
Fibrinogen Funcion [g/L]	2–4	38/4	3.8	0.75	35/7	3.66	0.66
INR	0.8–1.2	40/2	1.055	0.14	35/7	1.085	0.07
APTT [s]	23–35	40/2	30.3	5.3	35/7	29.15	4.7
APTT ratio	0.9–1.2	38/4	1.04	0.18	35/7	1.005	0.16
Thromb. intime	< 21	36/6	13.65	1.8	35/7	15.2	3

*NA data available from too few patients to calculate median

### Ethics approval

All procedures involving human participants have been approved according to the ethical standards of the institutional research committee, including the 1964 Helsinki Declaration and its later amendments of comparable ethical standards under number EK 29/2020. Ethics Committee of JFMED CU was registered under no. IORG0004721 at the US Office for Human Research Protection, US Department of Health and Human Services and after approval, the Committee got a certification with the code “IRB00005636 Jessenius Faculty of Medicine, Comenius University in Martin IRB # 1”.

### Consent to participate declaration

Informed consent for included participants was checked and approved by the ethical committees of Martin University Hospital and Jessenius Faculty of Medicine, Comenius University in Martin, and all signed informed consents have been archived for at least 20 years after the research was completed. 

### Control group

Blood samples from 42 subjects (20 male/ 22 female), median age 58 years, IQR 8.5 years, representing the normal population, were selected to match patients in age (± 2 years for each patient) and sex, with only a single mismatch in sex, in order to achieve a group as comparable as possible. The control individuals reported being subjectively healthy and not aware of any acute or chronic diseases, with no ischemic event in the history.

### Data processing

NMR-based metabolite profiling was performed on plasma samples following rapid centrifugation and methanol-based protein precipitation. Samples were reconstituted in deuterated buffer containing TSP-d_4_ as a chemical shift reference and measured on a 600 MHz Bruker Avance III spectrometer with TCI CryoProbe. 1D ^1^H NMR spectra were acquired using standard NOESY and CPMG pulse sequences, and selected 2D experiments (COSY, J-resolved) were recorded on a subset of samples for metabolite confirmation. Spectra were processed using Chenomx software and internal metabolite databases, with relative metabolite concentrations derived from well-resolved spectral regions. This NMR-based approach allows the simultaneous detection and quantification of multiple metabolites in a non-destructive and highly reproducible manner, making it suitable for systemic metabolic profiling. Its strengths include minimal sample preparation and robust quantification, while limitations include lower sensitivity for metabolites at very low concentrations and potential signal overlap in complex mixtures. Further methodological and statistical details, which were performed identically to our previous study (Duricek et al., 2025), can be found in that publication. Statistical analyses, paired Wilcoxon test and Mann Whitney U test were performed with multiple testing correction using the Benjamini–Hochberg false discovery rate (FDR) method.

#### Note

We use the trivial names of 2-oxoisocaproate – ketoleucine, 3-methyl-2-oxovalerate – ketoisoleucine, and 2-oxoisovalerate – ketovaline. We also use the abbreviation BCAAs for branched-chain amino acids: leucine, isoleucine, and valine, and BCKAs for their corresponding keto-derivatives.

MT patients refer to the group of patients who underwent mechanical thrombectomy.

## Results

### Multivariate and classification analyses

Altogether, 20 metabolites in blood plasma were evaluated, selected based on well-resolved peaks that could be reliably characterized: leucine, isoleucine, valine, ketoleucine, ketoisoleucine, ketovaline, alanine, glutamine, glucose, lactate, 3-hydroxybutyrate, pyruvate, succinate, citrate, lysine, creatine, tyrosine, histidine, phenylalanine, and tryptophan.

**Fig. 1:**
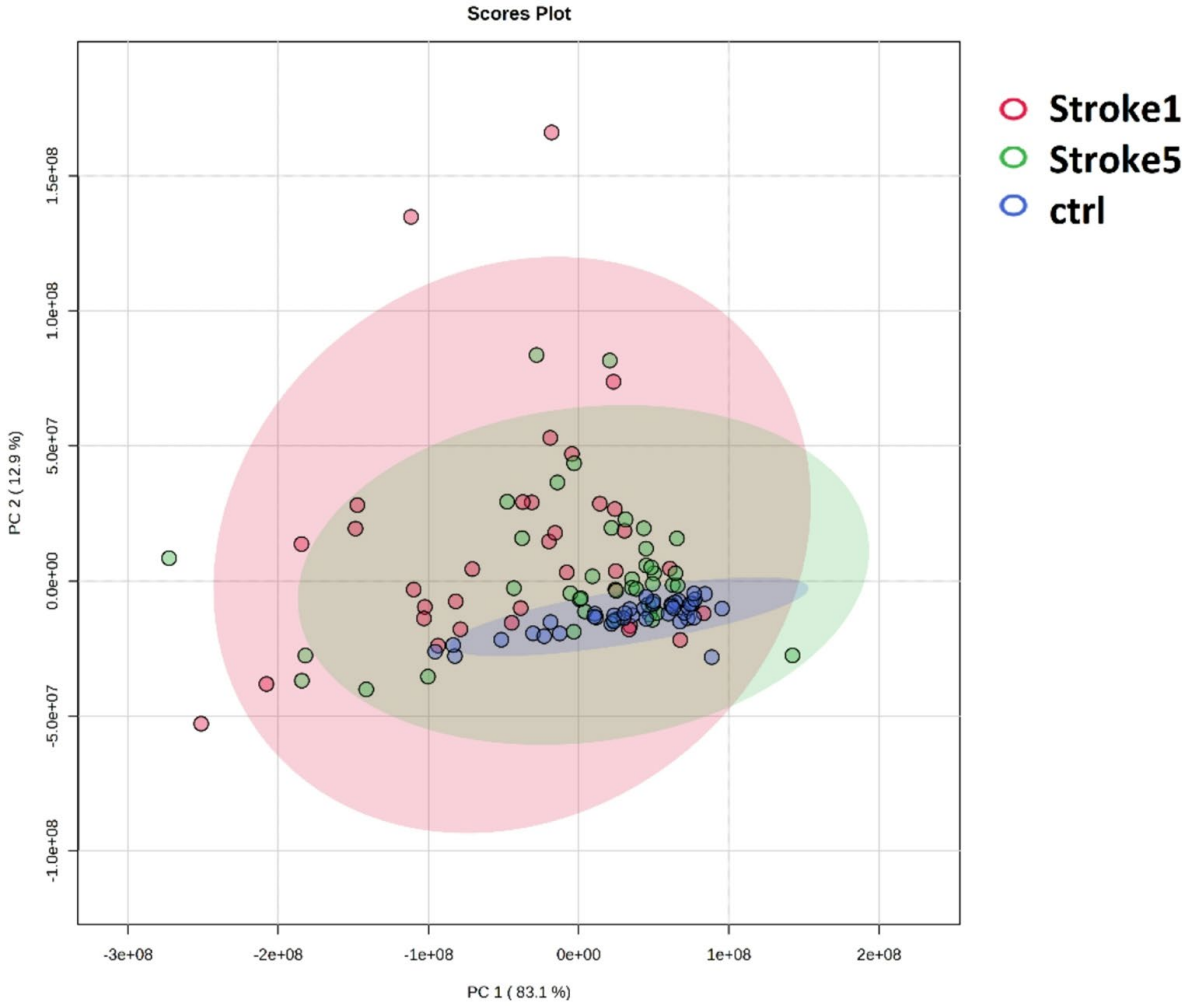
PCA analysis of metabolic data from blood plasma collected from Stroke patients on Day 1 (Stroke1) and on Day 5 after stroke (Stroke5) for the entire patient cohort, and the control group

Only metabolites meeting these criteria were included in the analysis to ensure accuracy and reproducibility of quantification. For PCA analysis, relative levels of blood plasma metabolites were used as input variables. As shown in Fig. 1, Stroke1 and Stroke5 patients exhibited greater spread within the 95% confidence interval ellipses compared to controls, suggesting substantially higher variability among patient samples. PC1 accounted for over 80% of the total variance, and together with PC2, they explained approximately 95% of the total variance.

Principal component analysis (PCA) is often followed by partial least squares discriminant analysis (PLS-DA) or related methods, which aim to maximize class separation. However, these methods are prone to overfitting, potentially producing overoptimistic results (Rodríguez-Pérez et al., 2018). To mitigate this, we employed a cross-validated Random Forest algorithm, which is less susceptible to overfitting and provides a more realistic estimate of discriminative performance. Similar to PCA, the input variables were the relative levels of metabolites in blood plasma. Discriminatory power was evaluated using accuracy, AUC (area under the ROC curve), and out-of-bag (OOB) error — estimated from the proportion of misclassified samples among those not used for training.

**Fig. 2:**
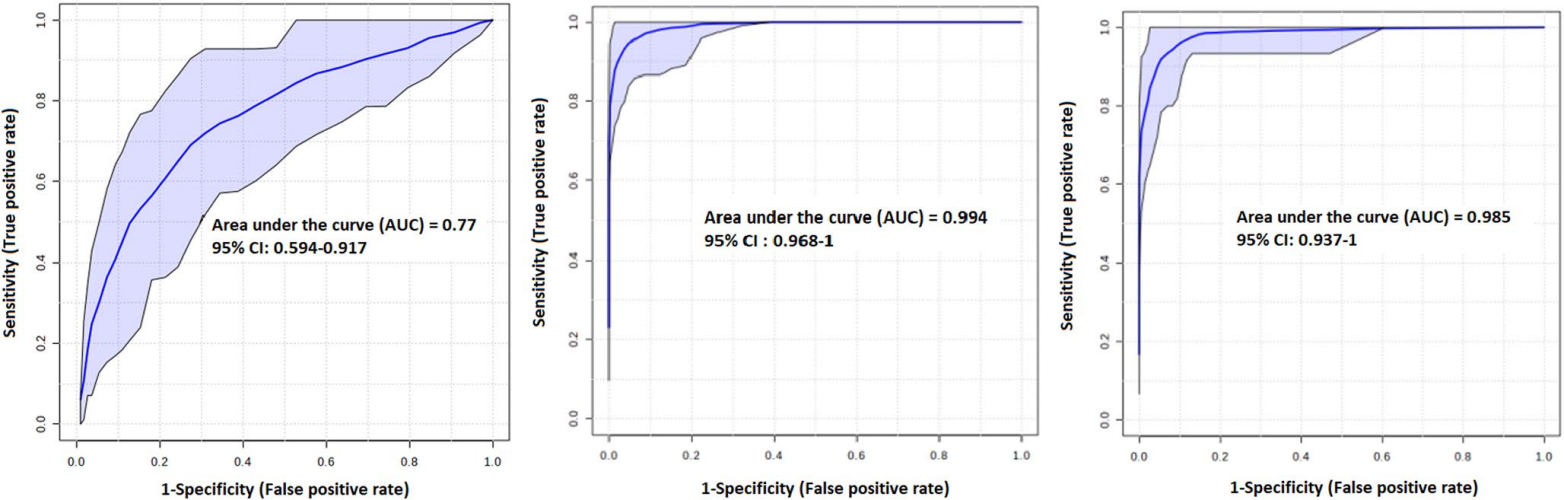
ROC curves for binary classifications: left, Stroke1 vs. Stroke5; middle, Stroke1 vs. controls; right, Stroke5 vs. controls. Relative levels of metabolites were used as input variables for the Random Forest algorithm

The metabolic differences between the first (Stroke1) and second (Stroke2) patient samples were sufficiently large to allow classification with over 70% accuracy. More relevant for practical applications is discrimination against the control group, representing the general population without acute ischemic stroke. Based on AUC values, this discrimination exhibited high performance. Classification accuracy was 94.6% for Stroke1 vs. control and 93.5% for Stroke5 vs. control, demonstrating high, though not perfect, classification performance across all samples (Fig. 2). The details of the discrimination analysis, including the five most important variables that contributed to these results, are shown in Table 3.

**Table 3:** Details of Random Forest discrimination for binary systems: relative levels of blood plasma metabolites from the entire patient cohort were used as input variables

Groups	AUC (95% CI)	OOB error	Predictive accuracy	Variables
Stroke1 – Stroke5	0.77 (0.594–0.917)	0.35	70.5%	Isoleucine, lactate, glucose, citrate, phenylalanine
Stroke1 - controls	0.994 (0.968-1)	0.09	94.6%	Glucose, ketoleucine, succinate, 3-hydroxybutyrate, phenylalanine
Stroke5 - controls	0.985 (0.937-1)	0.09	93.5%	Succinate, glucose, phenylalanine, isoleucine, ketoleucine

*AUC* area under the ROC curve, *CI* confidence interval, *OOB error* out-of-bag error. Results are displayed for the five most important variables

### Metabolic profiling

In the next section, we focused on understanding the impact of a cerebral ischemic event through the analysis of metabolite profiles. Following the Shapiro–Wilk test, the metabolic data were found to be predominantly non-normally distributed, and since normality cannot be reliably assessed in groups with fewer than 50 samples, we proceeded with non-parametric tests. The Mann–Whitney U test was used to compare metabolite levels between independent groups, while the paired Wilcoxon test was applied for paired samples (Stroke1 vs. Stroke5). Differences were observed in metabolites related to energy metabolism, including glucose, lactate, pyruvate, 3-hydroxybutyrate, citrate, succinate, and creatine; amino acids : BCAAs, alanine, glutamine, lysine, phenylalanine, tyrosine, tryptophan, histidine; and BCKAs. The data are summarised in Table 4 and are discussed in detail in the following section.

**Table 4:** Statistical evaluation of metabolic data from the entire patient cohort with FDR-adjusted p-value

	Stroke1 / Stroke5			Stroke1 / controls		Stroke5 / controls	
	pvalue	pvalue	Change	pvalue	Change	pvalue	Change
	Paired Wilcoxon test	Mann Whitney U test		Mann Whitney U test		Mann Whitney U test	
Leucine	0.0102	0.0109	-0.12	0.054	0.10	0.0000129	0.26
Isoleucine	0.00055	0.00053	-0.20	0.138	0.05	0.000000049	0.32
Valine	0.0438	0.0226	-0.09	0.79	-0.01	0.0067	0.09
Ketoisoleucine	0.225	0.455	-0.02	0.00042	-0.16	0.0081	-0.14
Ketovaline	0.552	0.67	0.01	0.0015	-0.13	0.0028	-0.14
Ketoleucine	0.192	0.441	-0.05	0.000000069	-0.28	0.0000126	-0.24
Alanine	0.798	0.95	-0.03	0.102	0.07	0.056	0.10
Glutamine	0.192	0.293	-0.05	0.036	0.08	0.00067	0.13
Glucose	0.0069	0.0129	0.40	0.000000013	0.77	0.00000168	0.26
Lactate	0.0063	0.0025	0.60	0.0000021	0.99	0.0137	0.24
3-Hydroxybutyrate	0.364	0.372	0.17	0.000000069	0.88	0.0000178	0.60
Pyruvate	0.192	0.352	-0.05	0.00049	0.44	0.00000026	0.52
Succinate	0.552	0.455	0.04	0.000000069	0.69	0.000000149	0.62
Citrate	0.0016	0.0058	0.23	0.000051	0.25	0.49	0.02
Lysine	0.0052	0.0095	-0.09	0.86	0.01	0.0028	0.11
Creatine	0.214	0.11	-0.07	0.0137	0.14	0.00000168	0.24
Tyrosine	0.0063	0.0333	-0.13	0.89	-0.02	0.0102	0.12
Histidine	0.192	0.309	-0.01	0.0036	-0.09	0.176	-0.09
Phenylalanine	0.0102	0.0109	-0.16	0.0000021	0.28	0.000000004	0.54
Tryptophan	0.171	0.186	-0.09	0.035	-0.14	0.63	-0.05
Phenylalanine / Tyrosine ratio	0.134	0.236	0.07	0.0000024	0.35	0.00000048	0.27

Changes were calculated as the difference between medians relative to the median of the comparison group

## Discussion

### Metabolic alterations in blood plasma

An increase in blood glucose levels following a cerebral ischemic event is a well-documented phenomenon that can occur in both diabetic and non-diabetic individuals. Acute hyperglycemia after ischemia has been associated with a higher risk of mortality and poorer functional outcomes (Capes et al., 2001). It has been identified as an independent predictor of increased 30-day mortality (Mi et al., 2017) and three-month mortality, regardless of diabetic status (Tsivgoulis et al., 2019). Dynamic changes in glycemia after stroke in humans cannot be generalized, as different patterns including persistent normoglycemia and transient, persistent, and delayed hyperglycemia were observed, influenced by various factors (Yong & Kaste, 2008). Stress-induced elevation of blood glucose, observed after an ischemic event, is not necessarily equivalent to diabetes mellitus-related hyperglycemia, although temporary relative insulin deficiency and increased rate of hepatic gluconeogenesis are major drivers of this condition (Vedantam et al., 2022).

In this study, as expected, we observed increased blood glucose levels in patients within 24 h after the onset of stroke symptoms, as determined by both clinical biochemistry (Table 1) and NMR analysis (Table 4). It should be emphasized that we excluded patients with type II diabetes mellitus and included only compensated type II diabetic patients who were not persistently hyperglycemic, so the detected increase in blood glucose levels can be attributed to the physiological response to cerebral ischemia. Five days after the stroke, circulating glucose levels decreased (Table 4), although they remained above those of the control group. Upon closer examination, patients who underwent mechanical thrombectomy (MT patients) exhibited higher blood plasma glucose levels following stroke compared to those without intervention, both on the first and fifth day. This finding is particularly interesting given that MT mitigates ischemic injury. However, clinical data revealed that patients in this study who underwent mechanical thrombectomy had moderate to severe neurological impairment (average NIHSS 13.5, range 10–18), which was notably higher compared to those without MT (average NIHSS 6.8, range 2–11). This evidence suggests a link between the metabolic response and the severity of neurological impairment following stroke, as discussed later.

After an ischemic event, the interrupted blood supply to the brain causes a lack of oxygen, accelerating anaerobic glycolysis, with an increased local production of lactate. The elevated lactate levels in the affected brain region after cerebral ischemia are well documented in experimental (Hoehn et al., 2001; Hoxworth et al., 1999) as well as in human studies (Combs et al., 1990; Henriksen et al., 1992; Saunders, 2000; Houkin et al., 1993). Post-ischemic increases in brain lactate during the reperfusion or reoxygenation period may serve as an alternative energy substrate and, via pyruvate, enter the tricarboxylic acid cycle to maintain ATP production as efficiently as glucose does. Many studies have proven the fact that lactate has beneficial effects in the recovery from damage caused by ischemic brain injury, as demonstrated in both animal models (Berthet et al., 2012; Berthet et al., 2009; Buscemi et al., 2022; Castillo et al., 2015; Rice et al., 2002; Roumes et al., 2021) and in human patients (Bouzat et al., 2014; Carteron et al., 2018; Ichai et al., 2013). When production of lactate exceeds local utilization, lactate is transported out of the brain into the bloodstream via monocarboxylate transporters (MCTs). Furthermore, factors related to stroke, such as severity, duration, and extent of damage, influence BBB integrity (Prakash & Carmichael, 2015), thereby affecting lactate release into circulation. Paradoxically, despite lactate being a hallmark metabolite of hypoxia, plasma lactate has not been consistently identified in clinical studies as a reliable predictor of outcomes following cerebral ischemic events. In human patients after stroke, elevated plasma lactate levels were observed within 72 h of the onset (Jung et al., 2011) and seven days after the onset of the ischemic event in serum (Wang et al., 2017), but many other experimental studies on cerebral ischemia including those from our laboratory did not confirm these results (Baranovicova et al., 2020; Baranovicova et al., 2018; Baranovicova et al., 2021; Baranovicova et al., 2022). The fact, that the unquestionable increase in tissue lactate after the ischemic injury is not always reflected in plasma can be explained by the assumption that the ischemic core is not necessarily the sole source of increased blood lactate after ischemia, and lactate changes in circulation also reflect a systemic metabolic response.

**Fig. 3:**
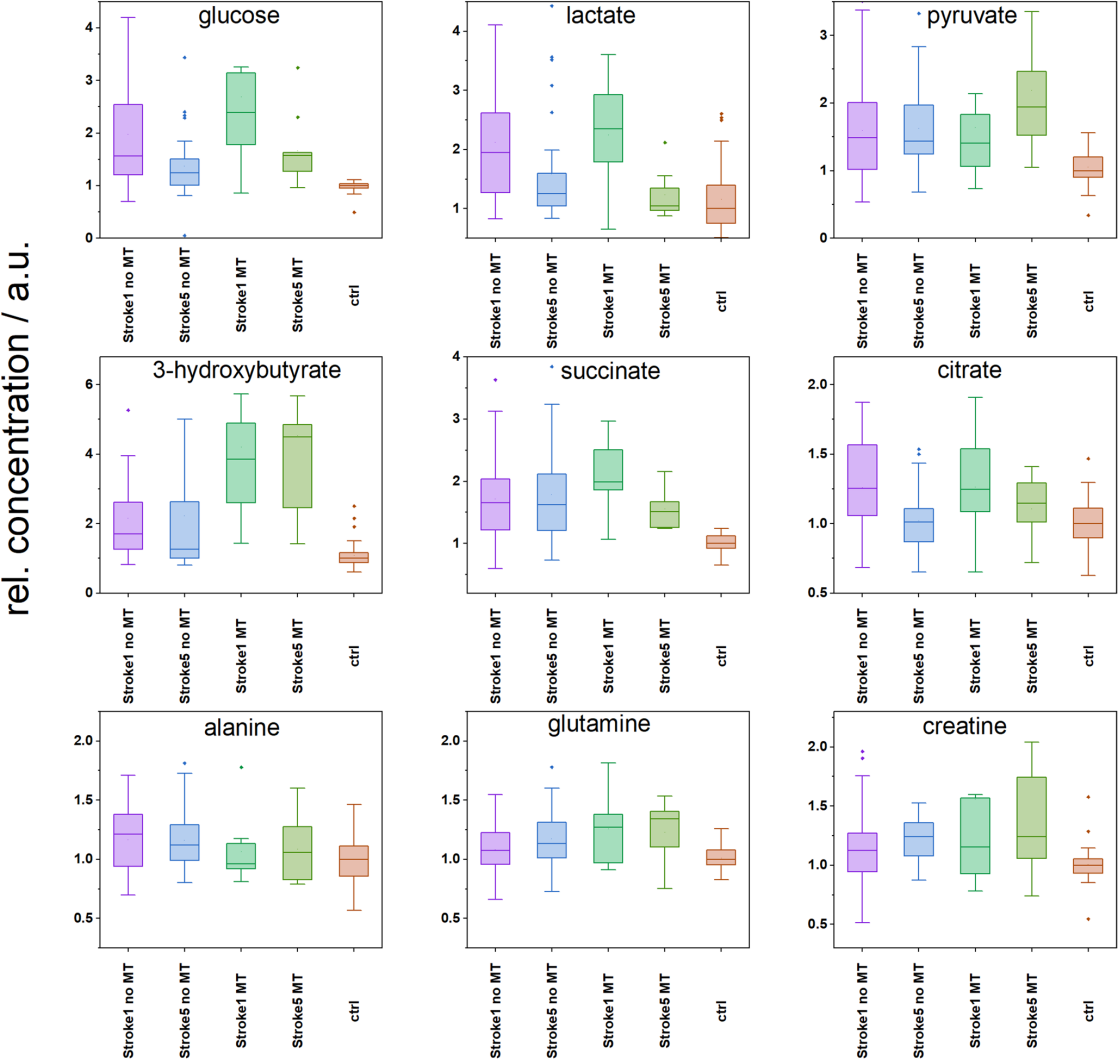
Relative levels of selected metabolites in patients after stroke are shown as follows: Stroke1 – the first day of hospital admission; Stroke5 – Dday 5 of hospitalization; MT – mechanical thrombectomy; and controls (ctrl), median of controls set to 1

In our study, elevated blood lactate levels were observed concurrently with increased blood glucose, and as circulating levels of glucose decreased on Day 5, circulating lactate levels also declined (Fig. 3). Blood lactate has gained considerable attention after studies revealed that the contribution of glucose to the TCA cycle is largely indirect, with circulating lactate substantially contributing to oxidative metabolism in multiple tissues (Hui et al., 2017; Rabinowitz & Enerbäck, 2020). In other words, the lactate produced via glycolysis is returned to circulation, transported via the bloodstream then taken up by secondary tissues where it is converted to pyruvate, which is oxidized to acetyl-CoA and further utilized in the TCA cycle. This illustrates the intriguing concept that inter-tissue lactate shuttling functionally separates glycolysis from the TCA cycle, enabling tissues to regulate these processes independently according to their metabolic demands.

During ischemic brain injury, in addition to other damaging mechanisms, excitotoxic glutamate inhibits mTOR signaling and causes neuronal insulin resistance (Pomytkin et al., 2019), making neurons more vulnerable to metabolic stress. This condition may result in metabolic reprogramming for energy substrates other than glucose to preserve cell viability. Following BBB disruption and impaired neural glycolysis after ischemia, alternative energy substrates such as ketone bodies may help meet metabolic demands during the reperfusion period. Under normal physiological conditions, ketone bodies can supply up to 70% of the brain’s energy, more energy efficient than glucose (White & Venkatesh, 2011). During acute brain injury, cerebral uptake of ketones has been shown to increase significantly (White & Venkatesh, 2011). This uptake not only stimulates cerebral blood flow but also leads to immediate oxidation of ketone bodies, which in turn reduces glucose utilization by the brain, even in sufficient glucose availability (Hasselbalch et al., 1996). Suzuki’s results indicated that 3-hydroxybutyrate, the main circulating ketone, is utilized as an energy substrate and may ameliorate disruptions in cerebral energy metabolism following hypoxia, anoxia, and ischemia, during which the anaerobic glycolytic pathway is activated (Suzuki et al., 2001). Furthermore, several studies have demonstrated that 3-hydroxybutyrate can protect neurons from glutamate-induced apoptosis and necrosis (Ziegler et al., 2003). Interestingly, ischemia-induced local lactate accumulation enhances the upregulation of monocarboxylate transporters (MCTs), which transport not only lactate (Contreras-Baeza et al., 2019) but also 3-hydroxybutyrate and other monocarboxylates (Halestrap, 2013). There is a legitimate question whether post-ischemic upregulation of MCTs by lactate can substantially increase the entry of 3-hydroxybutyrate into neuronal cells after stroke. Based on this, the results found in this study (Table 4; Fig. 3) can be combined as follows: Stroke induces hyperglycemia accompanied by increased levels of lactate and pyruvate. Lactate, serving as a shuttle energy source between tissues, could be utilized where needed, including brain tissue. Pyruvate, being a co-metabolite to lactate, accumulates concurrently, without being utilized in the amount produced. During significant metabolic shifts, the body produces 3-hydroxybutyrate to fulfill energy demand. The most interesting part is that both lactate and 3-hydroxybutyrate are of an increased concentration in the circulation, both of them may serve not only as an energy fuel for the damaged brain (accelerated entry enabled by upregulation of MCTs) but also as supporting agents, having proven strong neuroprotective effects on ischemically damaged neurons. Detailed analysis of our data revealed that MT patients exhibiting moderate to severe ischemic manifestations maintained substantially elevated circulating levels of 3-hydroxybutyrate on the fifth day after stroke. In contrast, patients without MT intervention, with lower NIHSS scores, showed a decline in 3-hydroxybutyrate levels at the same time point (Fig. 3). This observation may indicate that sustained elevation of 3-hydroxybutyrate in patients undergoing MT reflects a prolonged systemic metabolic stress response proportional to injury severity.

**Fig. 4:**
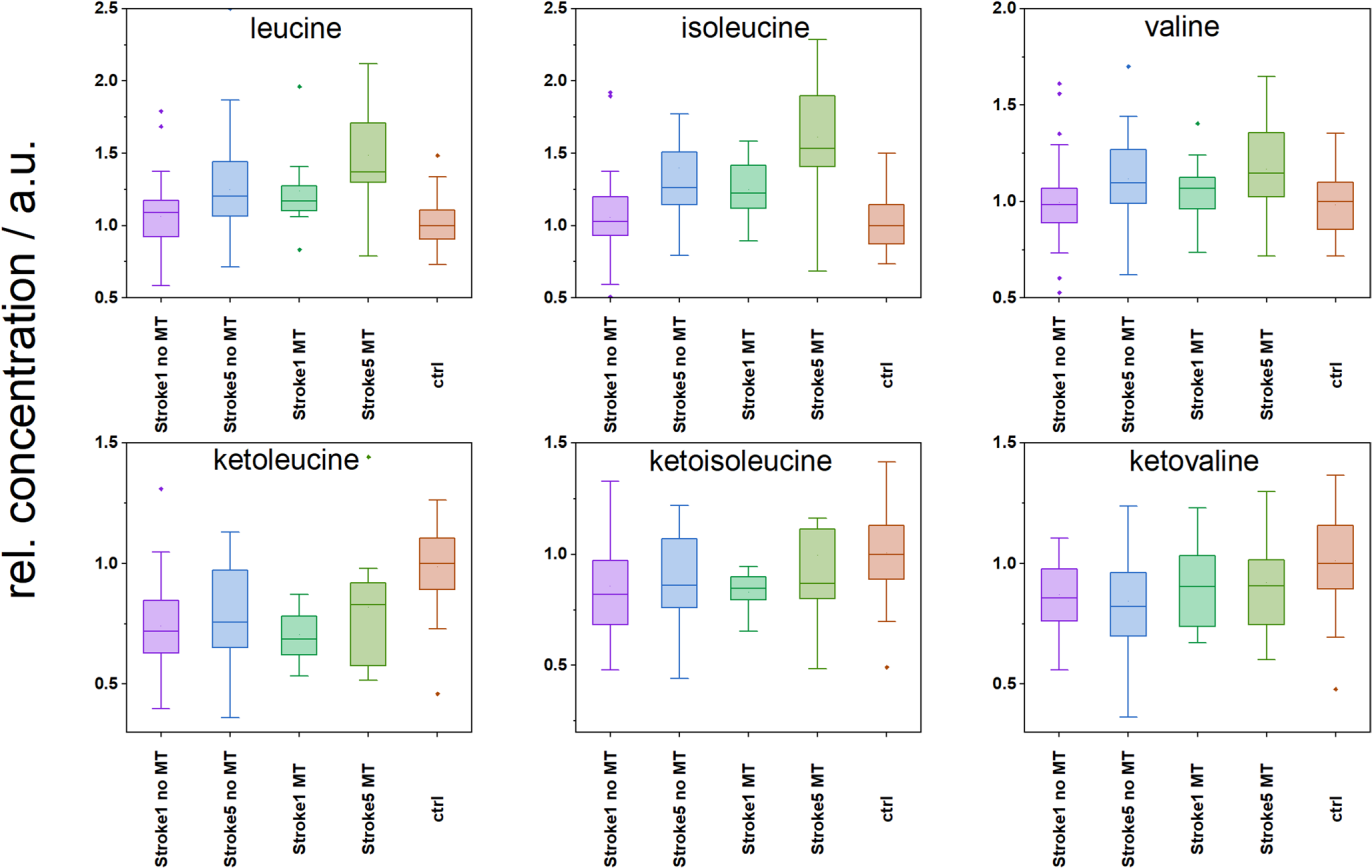
Relative levels of BCAAs and BCKAs in patients after stroke are shown as follows: Stroke1 – the first day of hospital admission; Stroke5 – Day 5 of hospitalization; MT – mechanical thrombectomy; and controls (ctrl), median of controls set to 1

Metabolism of BCAAs is known to be closely linked with glucose metabolism, the degree of insulin resistance and fatty acid metabolism (Holeček, 2018; Holeček, 2020). The exact mechanism is not well understood, as the previous studies reported variable data: BCAAs upregulate glucose transporters and activate insulin secretion (Zhang et al., 2017) whereas other authors suggested that BCAAs may induce insulin resistance through mTOR activation (White et al., 2016). In our previous animal studies, post-ischemic increases in blood plasma levels of BCAAs were not observed in the early phase (3 h after ischemia), but became evident 24 h after the cerebral ischemic event (Baranovicova et al., 2020; Baranovicova et al., 2018). In the patient cohort of the present study, BCAA levels were elevated only on the fifth day after stroke (Table 4); however, a more detailed analysis revealed a different pattern related to stroke severity: patients with severe ischemia exhibited elevated BCAA concentrations already on the first day (Fig. 4), which were much more pronounced on Day 5, whereas patients with less severe ischemic damage showed a significant rise only by the fifth day. These findings suggest that post-stroke BCAA elevations are both time- and severity-dependent.

The increased BCAA levels may have implications for the organism, especially in the context of an enhanced immune response. The data from Zhenyukh et al. suggest that high concentrations of BCAA could exert deleterious effects on circulating blood cells and therefore contribute to their proinflammatory and oxidative status (Zhenyukh et al., 2017), influencing the properties of microglia and their responsiveness to proinflammatory signals (Simone et al., 2013). Other studies have also indicated that inflammatory signals inhibit BCAA transport from the blood to muscles (Hasselgren et al., 1988; Holeček, 2018), which may further influence the blood plasma levels of BCAAs, as activation of immunocompetent cells after stroke develops gradually at multiple levels.

BCAAs can be reversibly deaminated to branched-chain keto acids (BCKAs) in muscle, where BCAA entry into muscle is dependent on insulin sensitivity (Bandt et al., 2022) and the rate of transamination responds rapidly to changes in tissue BCAA availability (Holeček, 2020). BCKAs, if not released back into the circulation, are irreversibly decarboxylated to yield their respective CoA compounds, which can serve as substrates for the TCA cycle. The metabolic pattern observed in post-stroke patients suggests that BCAAs accumulate in the bloodstream due to multiple factors, including reduced muscle uptake, insulin resistance, and cortisol-driven proteolysis, rather than a single mechanism, which may account for the BCKA depletion observed in this study (Fig. 4).

Both alanine and glutamine are major carriers of nitrogen, including ammonia, from extrahepatic tissues to the liver and play an important role in ammonia detoxification during periods of enhanced catabolism, trauma, or injury. In our study, we observed significantly increased levels of circulating alanine and glutamine, with glutamine showing an increasing trend on Day 5, more pronounced in MT patients. Previous studies have reported inconsistent results, with both elevated and decreased blood plasma levels of glutamine and alanine after cerebral ischemia (Ding et al., 2025; Kurtoğlu et al., 2025). The initial expectation might be decreased plasma glutamine levels, as it serves as a fuel for rapidly dividing cells, including immunocompetent cells, and lowered blood plasma glutamine is often observed during periods of activated immune response. However, during overproduction of acidic metabolites such as lactate and the ketone body 3-hydroxybutyrate — a state mimicking metabolic or lactic acidosis—additional processes are activated: the liver switches from a net consumer to a net producer of glutamine, which is then extracted by the kidney to help compensate for acidosis (Taylor & Curthoys, 2004).

In patients after stroke, we observed increased creatine levels, particularly on Day 5, which may reflect altered creatine and phosphocreatine metabolism in muscle and brain. The observed elevation in circulating creatine (Fig. 3; Table 4) may result from reduced physiological demand due to patients’ bed rest or from compensatory shifts in energy substrate utilization.

The observed shift in energy metabolism also affects TCA cycle functioning, as various energetic substrates are linked with the cycle at different phases. Fluctuations in TCA cycle intermediates, such as succinate and citrate, may be associated with stroke-induced mitochondrial dysfunction (Martínez-Fernández et al., 2001). Citrate is formed from acetyl-CoA and oxaloacetate in the presence of citrate synthase, representing the first reaction in the TCA cycle under intact conditions. Citrate levels were elevated in patient group within the first 24 h after stroke (Table 4). In patients who underwent MT, blood plasma citrate levels remained above control levels on the fifth day, whereas in patients with milder ischemic injury, circulating citrate levels normalized. Elevation of blood plasma citrate may reflect multiple factors, including alterations in mitochondrial oxidative metabolism, compensatory reliance on alternative substrates to maintain ATP production, cell damage, or changes in hepatic or renal metabolism in the early post-stroke period. In addition, disturbances in citrate homeostasis could also affect downstream metabolic pathways, including fatty acid synthesis and amino acid metabolism, highlighting the systemic impact of cerebral ischemia on cellular energy balance.

Both citrate and succinate are also linked to inflammation. The role of citrate in the production of acetyl-CoA for histone acetylation may be particularly important in regulating immune cell function (Williams & O’Neill, 2018). Succinate likely functions primarily as a signaling and messenger molecule (Wu, 2023), with its accumulation promoting inflammatory signaling (Mills & O’Neill, 2014). The mechanisms regulating succinate accumulation during the acute post-stroke inflammatory response likely involve a combination of multiple processes interfering at different levels, as reviewed by Mills and O’Neill (2014).

**Fig. 5:**
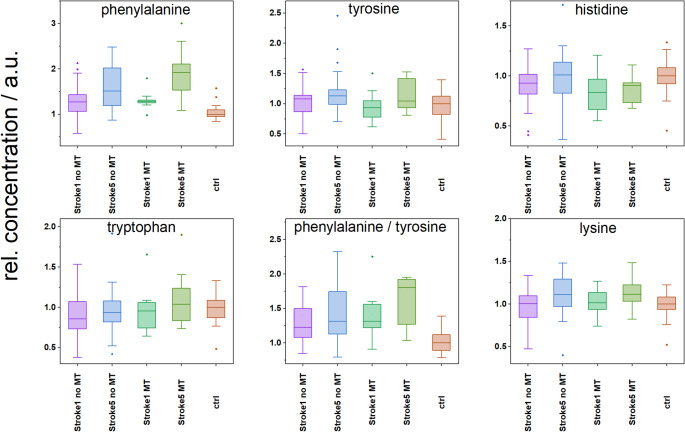
Relative levels of other evaluated metabolites in patients after stroke are shown as follows: Stroke1 – the first day of hospital admission; Stroke5 – Day 5 of hospitalization; MT – mechanical thrombectomy; and controls (ctrl), median of controls set to 1

Metabolic changes after a cerebral ischemic event also included increases in phenylalanine and tyrosine levels, particularly pronounced on Day 5 (Fig. 5). Results from Ormstad et al. (Ormstad et al., 2016) show that the Phe/Tyr ratio is highly elevated during the acute phase of ischemic stroke and that this elevation is associated with the inflammatory response. After recalculation, we also observed an increased Phe/Tyr ratio on Day 1, which became more pronounced on Day 5 (Fig. 5), particularly in MT patients, demonstrating, again, a higher metabolic impact of stroke in patients with more severe ischemic injury.

The final two metabolic changes reported in this study are decreased blood plasma levels of histidine and tryptophan. Histidine is an essential precursor of histamine, which plays a critical physiological role in the immune response by enhancing the activation of endothelial and immune cells and promoting the secretion of pro-inflammatory cytokines. However, evidence also suggests that histamine receptors are involved not only in immune cell activation but may also act as potent suppressors of inflammation (Akdis & Blaser, 2003). Tryptophan is an essential amino acid primarily metabolized via three main pathways: the kynurenine pathway, the indole pathway, and the serotonin/melatonin pathway. The kynurenine pathway is activated during the immune response following cerebral ischemia (Brouns et al., 2010) and may contribute to the depletion of tryptophan in blood plasma. A more detailed discussion is beyond the scope of this study, as the post-stroke immunological response is highly complex and comprehensive immunological data for our patients are lacking.

### Multivariate and classification analyses

The most notable difference, as shown by PCA, between the blood plasma metabolome of patients after stroke and controls (Fig. 1) was the considerable variability observed in stroke patients, suggesting an extensive metabolic response in multiple directions. For binary classification using the Random Forest (RF) classifier, relative metabolite levels in plasma were used as variables. The obtained AUC values were very close to 1, indicating nearly ideal performance in distinguishing Stroke1 and Stroke5 patients from controls (Fig. 2). As López-López and Barbas emphasized (López-López & Barbas, 2018), metabolites cannot be defined as biomarkers based solely on ROC analysis without proper clinical validation. For ROC curve generation, we used a cross-validated Random Forest algorithm, which mitigates overfitting by using approximately two-thirds of the data for training and the remaining portion for testing. This approach reduces bias from training and testing on the same dataset. While it does not replace the need for clinical validation, it provides promising insights in exploratory studies. A more detailed analysis showed predictive accuracy in the range of 93–94%, indicating that false classification occurred in just over 5% of the samples. Although this is encouraging, metabolomics covers only a relatively small subset of endogenous molecules compared to the number of genes, RNA species, or proteins. Therefore, in identifying potential low-molecular-weight biomarkers, some overlap between different pathologies can occur. Metabolites identified as the most imporatnt features, such as glucose and lactate, are associated with conditions like diabetes mellitus (DM) but are also part of a generalized metabolic response to many other stress conditions unrelated to stroke. Such overlap can complicate discrimination from populations other than healthy controls. Consequently, careful and critical assessment of the results is necessary.

### Notes to the study and its limitations

- The study was limited by a relatively small sample size and the exclusion of patients aged over 65 years, which affects the generalizability of our findings, meaing that the findings apply primarily to middle-aged stroke populations.
- All analyses were conducted in a single cohort without an independent validation group, which further limits the generalizability of the findings. Future studies should include larger, longitudinal cohorts with validation populations and standardized dietary and medication controls to confirm and extend these observations.
- The control group was recruited from the general population rather than a hospital setting, which may introduce selection bias. However, controls were screened for major metabolic diseases to minimize confounding.
- Another limitation of this study is also the lack of longitudinal data beyond Day 5 post-stroke. Longer-term follow-up was not feasible, as patients with milder deficits were typically discharged, while those with more severe injury were often transferred to other departments or long-term care facilities, precluding standardized follow-up measurements.
- Furthermore, there is a lack of dietary or medication control at the time of hospital admission in this study. Follow-up samples were collected in a fasting state, consistent with controls, but initial variability in diet or medication may have influenced the results.
- The presented results pertain to our study group, which was not subject to any selection criteria regarding lesion size or location, diagnostic or imaging findings, or other clinical variables. It is possible that with a larger sample size and more detailed subgroup analyses, the outcomes could provide deeper insights into metabolic profiles associated with specific clinical parameters. For example, we observed a positive correlation between post-stroke blood glucose levels (measured by both clinical biochemistry and NMR spectroscopy) and NIHSS score at admission (*p* = 0.03 for both Stroke1 and Stroke5 glucose values). However, this evaluation is based on a limited number of cases. Although our findings provide insight into the temporal and pathway-specific metabolic alterations after ischemic stroke, the direct link to functional recovery remains limited, and future studies with larger cohorts and longitudinal follow-up are needed to establish robust correlations between metabolite profiles and clinical outcomes.

## Conclusion

This study provides a comprehensive view of the dynamic metabolic response to acute ischemic stroke, revealing significant alterations in energy-related pathways, amino acid metabolism, and systemic metabolic regulation. The observed disturbances in glycolysis, elevated ketone body production, accumulation of TCA intermediates, and time-dependent changes in branched-chain and aromatic amino acids reflect the profound metabolic stress induced by cerebral ischemia. We demonstrated that the metabolic response to cerebral ischemia is very rapid for certain metabolites such as glucose and lactate, which almost normalized by the fifth day, whereas changes in other metabolites are more pronounced on Day 5, indicating a delayed metabolic response. Furthermore, stroke severity was shown to be an important factor affecting the extent of metabolic changes. The strong discriminatory power of plasma metabolite profiles at both time points highlights their potential for early diagnostic applications, although these findings should be critically assessed due to possible overlap with other pathologies.

Our findings underscore that post-stroke metabolic changes are not confined to isolated pathways but are interconnected through a complex network of biochemical interactions. This systemic metabolic communication may influence the trajectory of recovery as well as contribute to secondary pathophysiological processes, including potential impacts on peripheral organs. The partial normalization of some metabolite levels by Day 5 suggests early metabolic recovery which may reflect the initiation of compensatory mechanisms, although metabolic homeostasis was not fully restored. Overall, these results deepen our understanding of the systemic metabolic consequences of ischemic stroke and highlight the potential of metabolomic profiling as a tool for monitoring recovery after a cerebral ischemic event.

## Data Availability

The data supporting the findings of this study, including raw NMR spectra, are available upon reasonable request from the authors eva.baranovicova@uniba.sk, peter.racay@uniba.sk. All data were generated as part of this study, and no third-party or publicly available datasets were used.
